# Analytical Evaluation of Visby Medical RT-PCR Portable Device for Rapid Detection of SARS-CoV-2

**DOI:** 10.3390/diagnostics11050813

**Published:** 2021-04-29

**Authors:** Adriana Renzoni, Francisco Perez, Marie Thérèse Ngo Nsoga, Sabine Yerly, Erik Boehm, Angèle Gayet-Ageron, Laurent Kaiser, Manuel Schibler

**Affiliations:** 1 Laboratory of Virology, Laboratory Medicine Division, Diagnostic Department, Geneva University Hospitals, CH-1211 Geneva, Switzerland; Sabine.Yerly@hcuge.ch (S.Y.); Erik.Boehm@hcuge.ch (E.B.); Laurent.Kaiser@hcuge.ch (L.K.); Manuel.Schibler@hcuge.ch (M.S.); 2 Faculty of Medicine of Geneva, University of Geneva, CH-1211 Geneva, Switzerland; Francisco.PerezRodriguez@hcuge.ch (F.P.); Marie-Therese.NgoNsoga@hcuge.ch (M.T.N.N.); 3 Division of Infectious Disease, Geneva University Hospitals, CH-1211 Geneva, Switzerland; 4 CRC & Division of Clinical-Epidemiology, Department of Health and Community Medicine, University of Geneva & University Hospitals of Geneva, CH-1211 Geneva, Switzerland; Angele.Gayet-Ageron@hcuge.ch

**Keywords:** SARS-CoV-2, rapid diagnostic techniques, POCT techniques

## Abstract

Extended community testing constitutes one of the main strategic pillars in controlling the COVID-19 pandemic. Reverse transcription PCR (RT-PCR) targeting the SARS-CoV-2 genome on nasopharyngeal swab samples is currently the reference test. While displaying excellent analytical sensitivity and specificity, this test is costly, often requires a substantial turnaround time, and, more importantly, is subject to reagent and other material shortages. To complement this technology, rapid antigen tests have been developed and made available worldwide, allowing cheap, quick, and decentralized SARS-CoV-2 testing. The main drawback of these tests is the reduced sensitivity when RT-PCR is the gold standard. In this study, we evaluate Visby an innovative, portable, easy-to-use RT-PCR point-of-care (POC) diagnostic device. Our retrospective analysis shows that overall, compared to the Cobas 6800 RT-qPCR assay (Roche), this RT-PCR POC technology detects SARS-CoV-2 RNA with 95% sensitivity (95%CI = 86.3–99%) and 100% specificity (95% CI = 80.5–100%). For samples with cycle-threshold values below 31, we observed 100% sensitivity (95% CI = 66.4–100%). While showing an analytical sensitivity slightly below that of a standard RT-qPCR system, the evaluated Visby RT-PCR POC device may prove to be an interesting diagnostic alternative in the COVID-19 pandemic, potentially combining the practical advantages of rapid antigen tests and the robust analytical performances of nucleic acid detection systems.

## 1. Introduction

Complementary to prevention measures, such as social distancing, mask wearing, and hand hygiene, extensive population screening with isolation of positive cases is one of the key means of controlling the COVID-19 pandemic caused by severe SARS-CoV-2 [1]. SARS-CoV-2 RNA detection by RT-qPCR) in nasopharyngeal respiratory samples is currently considered to represent the gold standard for COVID-19 diagnosis. This technology, known for its high analytical sensitivity and specificity, has been applied to clinical virology diagnostics since the 1990s. On the other hand, it is costly, requires complex laboratory infrastructure, often has a high turnaround time, and even more importantly, RT-qPCR reagents and plastic materials are subject to shortages in the current crisis [2].

Rapid antigen tests are increasingly widely used, allowing for user-friendly, low-cost, quick, and decentralized testing. The main limitation of these tests is the reduced sensitivity in comparison to RT-qPCR, around 90% in the best-case scenario. Innovative nucleic acid detection point-of-care (POC) diagnostic technologies are now being developed, potentially combining the excellent analytical performance of RT-qPCR and the practical advantages of rapid antigen tests [3,4].

An alternative to the detection of antigen is the amplification of nucleic acid by reverse transcription in combination with isothermal methods such as: loop-mediated isothermal amplification (RT-LAMP) [5,6,7], recombinase polymerase amplification (RT-RPA) [8,9,10], and nicking-endonuclease amplification reactions (RT-NEAR) [11,12,13,14]; which do not need a thermal cycler, potentially enabling their use at POC facilities. The reference standard of RT-PCR also detects viral nucleic acid, and some devices capable of RT-PCR have been developed that are suitable for POC use, such as the DNAnudge Covid-19 test.

Recently, Visby Medical Inc. (Visby) has developed and begun manufacturing small palm-sized RT-PCR devices capable of detecting SARS-CoV-2. This device contains all necessary reagents, and is operated simply by loading a sample and sequentially pushing buttons; thus representing a self-contained easy-to-use RT-PCR POC diagnostic device. The device makes use of a continuous flow serpentine PCR channel combined with an enzyme linked detection chemistry to produce a colorimetric signal that is easily observable by the user. The device, originally developed for detection of bacterial targets in a sexually transmitted infection panel [15] has been adapted for rapid RT-PCR identification of the SARS-CoV-2 N gene and has received an FDA EUA for use in high and moderate complexity settings. More recently, on 8 February 2021, the Visby test received EUA for use in CLIA-waived settings (https://www.fda.gov/media/145917/download, accessed on 28 April 2021). Of note, the device may only be used once before requiring disposal or refurbishment by the manufacturer. Visby is intended for testing individuals suspected of COVID-19 by their health care provider using nasopharygeal, anterior nasal or mid-turbinate swabs collected into transport media,

In this study, we tested the new RT-PCR POC Device developed by Visby to detect the presence of SARS-CoV-2 RNA in a retrospective study using frozen SARS-CoV-2 positive nasopharyngeal samples using Cobas 6800 RT-qPCR assay (Roche) as the gold standard.

## 2. Material and Methods

### 2.1. Patient Samples and Assay Controls

According to the Swiss Ethics Committee on research involving leftover clinical samples, this study occurred in the context of a method quality validation in an emergency setting and therefore did not require any authorization from our ethics committee. The vast majority of positive samples come from outpatient symptomatic patients enrolled in a COVID-19 screening center at the Geneva University Hospitals. Sixty-one confirmed positive SARS-CoV-2 nasopharyngeal swab (NPS) samples were tested. Sample positivity, that is samples with a cycle-threshold (Ct) value below 35, was previously determined by using the Cobas 6800 SARS-CoV-2 RT-PCR assay (Roche, Switzerland) and stored in Copan UTM^TM^ or an in-house DMEM-based media that has been validated in our institution [16]. Samples were collected around 6 weeks or less prior to device testing and frozen at −80 °C. Patient specific data include demographics, symptom severity, and date of symptoms onset. Seventeen SARS-CoV-2 PCR negative NPS samples were tested as negative controls. Two external positive and negative controls (NATSARS (COV2)-ERC and NATSARS (COV2)-NEG, respectively) were obtained from ZeptoMetrix (Distributed by Helvetica Health Care, Geneva, Switzerland). External controls were performed when different operators were running the experiments. In total, 17 samples were considered as controls in this study.

### 2.2. Visby Medical POC Device Testing

**Figure 1:**
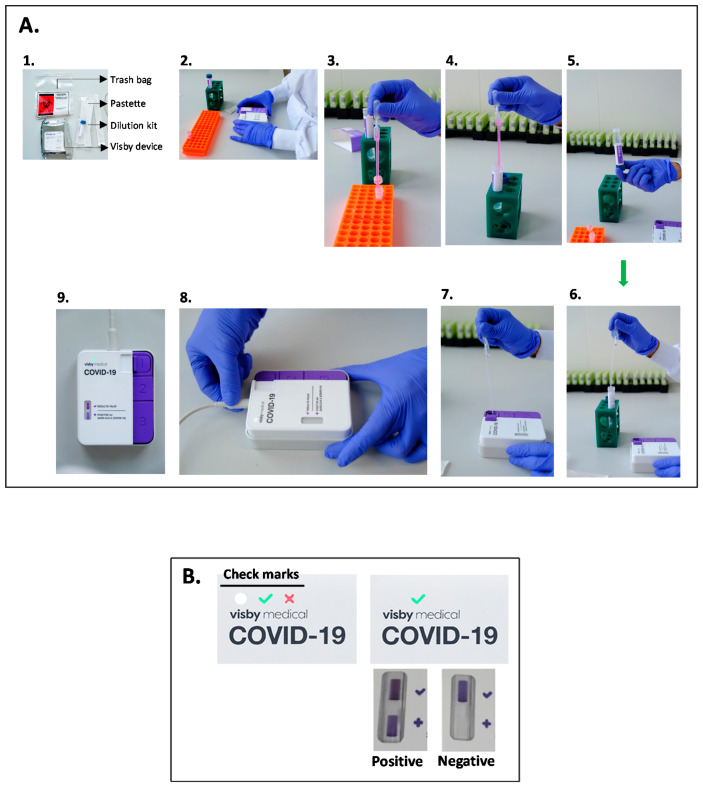
(**A**) Schematic steps for Visby Medical kit procedure. (1) Kit contents. (2) Patient sample preparation, with dilution buffer and cassette. (3–5) The sample is put into the dilution buffer and mixed by inversion. (6–7) 650 uL of the diluted sample is inserted into the device using the pastette. (8) Buttons 1, 2, and 3 are pushed, and the device is connected to electrical power. (9) Results displayed. A white checkmark appears, indicating that the test is in progress (see description below). (**B**) The left panel shows the white or green or red check marks denoting correct power and processing of the device, a valid test result ready to be read, or an invalid result due to an electrical error, respectively. The right panel shows an example of a valid (green light) and positive (visible upper and lower purple spots) or negative (only the upper purple spot is visible).

Analysis of samples and external controls was performed following the manufacturer protocol (https://www.fda.gov/media/142228/download, accessed on 28 April 2021) with some modifications, as we performed a retrospective study using frozen SARS-CoV-2 samples. Briefly, specimens were mixed by inversion 5 times, and 650 µL was inserted into provided dilution buffer using the special Pasteur pipette provided with the device (Visby Medical) (Figure 1A). The diluted sample was mixed five times by inversion, 650 µL was taken with a second Pasteur pipette and dispensed immediately into the device (Button No. 1). After loading the sample; sample extraction, reverse transcription, PCR amplification, and result visualization were started by sequentially pushing down buttons 1, 2 and 3 and plugging the device in. A successful device start is indicated by a white light. After 30 min, a green or red check mark appears confirming a valid or invalid device performance, respectively (Figure 1B). Samples run in invalid devices were retested using a new device.

Results of valid devices were displayed in the test result window as visible purple marks (Figure 1B). A purple mark in the upper position (indicated by a “√” symbol) indicates a valid sample extraction and amplification reaction, which was determined by detecting the human 18S ribosomal structural RNA present in nasopharyngeal samples. A purple mark in the lower position (indicated by a “+” symbol) indicates a positive amplification of SARS-CoV-2 nucleic acid. Visualization of both upper and lower position purple marks denotes a positive SARS-CoV-2 sample, while visualization of a single upper position purple mark denotes a negative SARS-CoV-2 sample (Figure 1B). The external positive and negative controls were used to ensure that test reagents are working correctly. Each control was run when a new operator was running the tests. Results interpretation was performed in a blinded manner by two laboratory staff members.

### 2.3. Comparison RT-qPCR Assay

The validated Roche Cobas 6800 SARS-CoV-2 RNA extraction and RT-qPCR assay were used as a reference assay (Cobas SARS-CoV-2 Ref 09175431190; Cobas SARS-CoV-2 Control kit Ref: 09175440190; Cobas 6800/8800 Buffer Negative Control kit Ref 07002238190; Roche, Switzerland). In case of discrepant results between the Cobas and Visby assays, viral RNA genome detection was also performed using GeneXpert (Ref: XPRSARS-CoV2-10 from Cepheid). Cobas targets ORF1a/b and a pan-Sarbecovirus conserved region of the E gene, while GeneXpert targets N and E genes.

### 2.4. Evaluation of Analytical Performances

The sensitivity and specificity of the device were determined by comparison to the Cobas assay, according to sensitivity = TP/(TP + FN); Specificity = TN/(TN + FP); where TP = true-positives, FN = false-negatives, TN = true-negatives, and FP = false-positives. Confidence intervals of 95% (95% CI) were calculated using the Clopper–Pearson method [17]. We reported overall sensitivity and specificity, as well as sensitivity by delay from symptoms onset and by categories of Ct values.

### 2.5. Limit of Detection (LoD)

As recommended by the manufacturer, a SARS-CoV-2 negative nasopharyngeal cell matrix was prepared to perform LoD analyses. First, nasopharyngeal samples, which were previously verified to be SARS-CoV-2 negative, were pooled to obtain a total of 12 mL. The pooled nasopharyngeal sample was retested by Cobas RT-qPCR to ensure that the sample pool was negative and immediately frozen at −80 °C. SARS-CoV-2 viral stock was produced as follows: SARS-CoV-2 isolates were collected from ex vivo infections of airway epithelia cultured in an air-liquid interface as previously described [18]. Briefly, after 3 h of apical virus inoculation, in vitro differentiated respiratory tissues (MucilAir^TM^, Epithelix SARL, Geneva, Switzerland) were washed three times with PBS (Phosphate Buffered Saline, Sigma, St. Louis, MO, USA) and incubated in MucilAir^TM^ medium at 33 °C and 5% CO_2_. Four days post infection, respiratory tissues culture supernatant is removed and 200µL of MucilAir^TM^ medium was added apically and viral culture supernatant was collected after 20 min of incubation at 33 °C at 5% CO_2_. Viral load in the culture supernatant was determined by RNA quantification. Briefly, RNA was extracted with NucliSens easyMAG (bioMérieux, Marcy-l’Étoile, France), and quantified by RT-qPCR using SuperScript™ III Platinum™ One-Step qRT-PCR Kit (Invitrogen, Carlsbad, CA, USA) in a CFX96 Thermal Cycler (Bio-Rad, Hercules, CA, USA). RT-qPCR was performed using a specific set of primers and probes targeting the SARS-CoV-2 E gene (forward primer: 5′-ACAGGTACGTTAATAGTTAATAGCGT-3′, reverse primer: 5′-ATATTGCAGCAGTACG CACACA-3′, and the probe: 5′-6-FAM- ACACTAGCCATCCTTACTGCGCTTCG-BBQ-3′) [19]. In vitro transcribed SARS-CoV-2 E gene RNA (EVAg, Essen, Germany) was used as a reference standard to convert Ct values into RNA copies/mL. Data were analyzed using Bio-Rad CFX maestro software (Bio-Rad, Essen, Germany). Quantified culture supernatant was finally diluted in PBS to a concentration of 2.1 × 10^6^ copies/mL.

## 3. Results

### 3.1. Visby POC Device and Cobas SARS-CoV-2 Nucleic Acid Detection Concordance

Frozen samples were thawed, dispensed immediately into dilution buffer provided with the device, and further processed to avoid nucleic acid damage as much as possible. After 30 min of sample processing, purple mark recording was performed immediately in devices displaying the green light. An invalid run occurred for a single sample, but after retesting, yielded a valid result.

**Figure 2:**
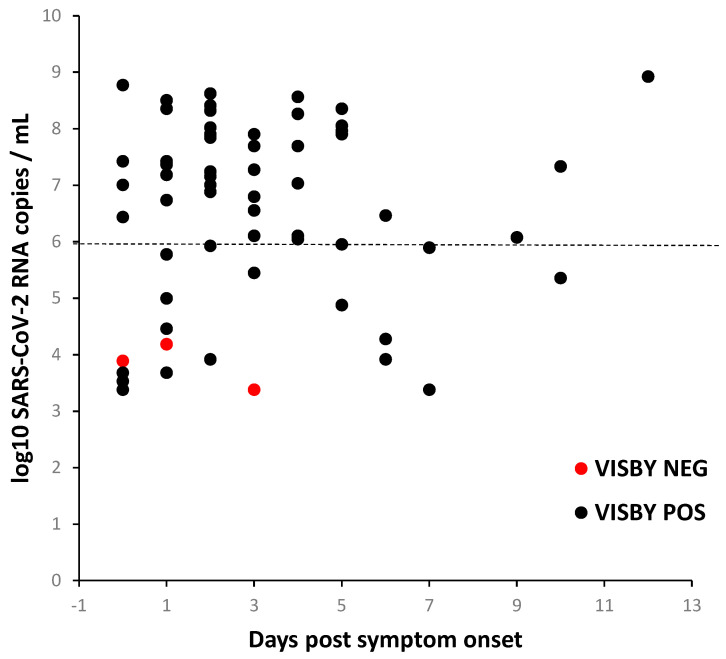
Detection of SARS-CoV-2 by Visby device based on viral loads and the time of symptom onset. SARS-CoV-2 viral loads by the time of symptom onset for the symptomatic and RT-qPCR positive individuals. Black and red dots represent positive or negative Visby results, respectively. Dotted line: 6 log_10_ SARS-CoV-2 RNA copies/mL shows the VL threshold limit determining the number of culturable viruses. Note: Samples classified at day 0 include 4 samples with unknown dates of symptoms.

The clinical accuracy of the device compared to Cobas RT-qPCR was calculated. From the 61 Cobas-positive samples (Ct values from 15.5 to 34), the device detected 58 positive samples with 3 false-negative samples. All 17 Cobas-negative samples were also found to be negative by the device (Table 1). We observed a sensitivity of 95.1% (CI 95% = 86.3–99%) and a specificity of 100% (CI 95% = 80.5–100%). An analysis by Ct values showed that the device achieved 100% sensitivity in samples with Ct values below 35 (Table 2). As shown in Table 3, the device achieves > 95% sensitivity with tests performed from 2 days after onset of symptoms, and 100% (73.5–100%) from 4 days post symptoms onset. We further evaluated Visby results in relation to viral loads and days post onset of symptoms, and observed that the few false-negative results were observed with three samples with low viral loads (Figure 2).

Since discrepant results might be explained by the effects of sample freezing on RNA stability, we retested the discordant samples with the Cobas assay. Nearly identical positive Cobas Ct values were found, showing that RNA degradation upon thawing samples could not explain the negative device results (Table S1). Discrepant sample results might be explained by detection of different genes used by the device (N-gene) and the Cobas (E and Orf1 genes) assay. We therefore analyzed discrepant samples with the SARS-CoV-2 GeneXpert assay, targeting N and E genes, however, both genes were detected (Table S1).

### 3.2. Analytical Limit of Detection (LoD)

A clinical SARS-CoV-2 isolate was cultured in an air-liquid interface respiratory epithelium system (mucilAir). Viral supernatant concentration was quantified using E-gene standards from the European virus archive and diluted to a final concentration of 2.1 × 10^6^ copies/mL. Live virus from cell culture supernatant were serially diluted (10^5^, 10^4^, 10^3^, 10^2^, 10^1^ copies/mL) in SARS-CoV-2 negative nasopharyngeal cell matrix to evaluate the LoD. We tested a single replicate of each dilution and found them all positive, showing a Visby positive detection with low viral loads (10^2^ copies/mL). Our data are in agreement with the manufacturer LoD determination, which showed a 95% detection rate for 1112 genomic copies/mL and a detection rate of 45% for 125 genomic copies/mL (Visby medical package insert).

## 4. Discussion and Conclusions

The aim of this study was to evaluate the Visby POC device to detect the presence of SARS-CoV-2 RNA in previously collected frozen clinical nasopharyngeal samples, in comparison to the Cobas 6800 RT-qPCR assay (Roche). The devices detected SARS-CoV-2 RNA with 95% sensitivity and 100% specificity.

Few discrepant results were observed that might be explained by potential N-gene mutations that are undetectable by the device, similarly to previously published observations showing that failure to detect SARS-CoV-2 by the Cobas 6800 assay was linked to E-gene mutations [20]. This is a possible scenario, but an unlikely one, as extensive in silico analysis of variants were detected by Visby primers and probes (https://www.fda.gov/media/142228/download, accessed on 28 April 2021). Having excluded potential issues related to different viral genes targeted with both assays, the reduced sensitivity observed with the devices probably results from the different technologies applied, which differ in technical details such as buffers and sample processing methods. Visby’s test is not directly compatible with most viral transport media (manufacturer package insert). While dilution of samples collected in transport media mitigates the inhibitory effects of the transport media, it reduces the test sensitivity, which could account for the 3 Cobas-positive samples that the device missed. However, this dilution step did not prevent detection of other samples with similar viral loads. We cannot exclude the possibility of PCR inhibiting substances within these nasal samples that prevent gene amplification exclusively with the device methodology [21].

Dilution of the sample collected into transport media to mitigate the inhibitory effects of the transport media may reduce the sensitivity of the test. While a remnant sample evaluation was performed in this study, it is reasonable to assume that direct swab elution with no dilution step would yield an improvement in sensitivity. A prospective clinical study would be important to assess the clinical sensitivity of the direct swab method.

In the present analytical study, the POC Device performs less well at Ct values ≥35; however, the reduction in sensitivity is relatively unimportant since high Ct values probably indicate a low transmission risk [22]. Furthermore, the sensitivity displayed by the device is still substantially higher than that of the 85% to 90% of the best antigen tests [23,24,25,26,27].

This device operates by detecting viral nucleic acids such as the reference standard (RT-PCR) and isothermal amplification tests. RT-LAMP amplification unfortunately has issues such as: non-specific amplification that can be a problem in later steps as a result of “carry-over contamination”, “product cross contamination” [28,29,30], or primer hybridization [31].

In contrast, the device evaluated here uses the same robust and well established principle used by the gold-standard tests, and thus is expected to be reliable and accurate. Other RT-PCR devices suitable for POC diagnostics are on the market (i.e., NudgeBox or LIAT) but are less portable than the Visby POC device evaluated here, and may be unavailable or have a sample processing time of up to 90 min that may be prohibitive for some envisioned POC uses [4].

It is important to note the limitations of our study. The selection of samples tested is not representative of an epidemiological trend because only frozen samples were used, and no prospective recruitment of patients was performed with both methods concomitantly. No estimation of positive or negative predictive values can be performed as samples were not consecutively collected and prevalence is not interpretable. Future clinical evaluations, including head-to-head comparisons with antigen tests and isothermal tests, testing of SARS-CoV-2 asymptomatic-contacts, and screening healthcare workers will help to better position SARS-CoV-2 nucleic acid detection among the POC diagnostics.

In summary, the Visby device appears to be an attractive alternative POC test, performing nearly as well as conventional RT-qPCR tests, while displaying the appealing characteristics of a POC test. It is available in a portable format with minimal requirement of technical skills. After sample acquisition, less than 1 min is needed for treatment and loading samples onto de VISBY device. Viral extraction, amplification and easy-readout are obtained after exactly 30 min. This short turnaround time allows for rapid patient isolation or orientation decisions, e.g., in nursing homes or even hospital settings lacking more complex laboratory equipment. A potential disadvantage includes the generation of waste that remains an issue to be addressed. The company is exploring recycling components to reduce generated waste.

## Figures and Tables

**Table 1:** Sensitivity and specificity of the Visby device.

	Reference RT-qPCR Cobas POS	Reference RT-qPCR Cobas NEG	Total
Visby POS	58	0	58
Visby NEG	3	17	20
TOTAL	61	17	78

Sensitivity: 95% (95% CI 86–99)/specificity: 100% (95% CI 80.5–100).

**Table 2:** Sensitivity of Visby device depending on RT-qPCR CT values.

CT Values Cut-Offs	Sensitivity	95% CI	N
15–20	100%	82–100%	19
21–25	100%	84–100%	21
26–30	100%	66–100%	9
31–35	100%	43–94.5%	12

**Table 3:** Sensitivity of Visby device by the delay since symptom onset.

Delay Since Onset (Days)	Sensitivity	95% CI	N
0	87.5%	47.3–99.7%	8
1	90.9%	58.7–99.8%	11
2–3	95.2%	76.2–99.9%	21
4–5	100%	73.5–100%	12
6–7	100%	47.8–100%	5
>7	100%	39.8–100%	4

## Supplementary Materials

The following are available online at https://www.mdpi.com/article/10.3390/diagnostics11050813/s1. Table S1: Positive and negative RT-qPCR Cobas samples tested on RT-PCR Visby diagnostic device. C = corncordant result; NC = Non-concordant results. INV = invalid result.

## References

[1] Udugama B., Kadhiresan P., Kozlowski H.N., Malekjahani A., Osborne M., Li V.Y.C., Chen H., Mubareka S., Gubbay J.B., Chan W.C.W. (2020). Diagnosing COVID-19: The Disease and Tools for Detection. ACS Nano.

[2] Dinnes J., Deeks J.J., Adriano A., Berhane S., Davenport C., Dittrich S., Emperador D., Takwoingi Y., Cunningham J., Beese S. (2020). Rapid, point-of-care antigen and molecular-based tests for diagnosis of SARS-CoV-2 infection. Cochrane Database Syst. Rev..

[3] Joung J., Ladha A., Saito M., Segel M., Bruneau R., Huang M.W., Kim N.-G., Yu X., Li J., Walker B.D. (2020). Point-of-care testing for COVID-19 using SHERLOCK diagnostics. medRxiv.

[4] Gibani M.M., Toumazou C., Sohbati M., Sahoo R., Karvela M., Hon T.-K., De Mateo S., Burdett A., Leung K.Y.F., Barnett J. (2020). Assessing a novel, lab-free, point-of-care test for SARS-CoV-2 (CovidNudge): A diagnostic accuracy study. Lancet Microbe.

[5] Notomi T., Okayama H., Masubuchi H., Yonekawa T., Watanabe K., Amino N., Hase T. (2000). Loop-mediated isothermal amplification of DNA. Nucleic Acids Res..

[6] Taki K., Yokota I., Fukumoto T., Iwasaki S., Fujisawa S., Takahashi M., Negishi S., Hayasaka K., Sato K., Oguri S. (2021). SARS-CoV-2 detection by fluorescence loop-mediated isothermal amplification with and without RNA extraction. J. Infect. Chemother..

[7] Nawattanapaiboon K., Pasomsub E., Prombun P., Wongbunmak A., Jenjitwanich A., Mahasupachai P., Vetcho P., Chayrach C., Manatjaroenlap N., Samphaongern C. (2021). Colorimetric reverse transcription loop-mediated isothermal amplification (RT-LAMP) as a visual diagnostic platform for the detection of the emerging coronavirus SARS-CoV-2. Analyst.

[8] Xia S., Chen X. (2020). Single-copy sensitive, field-deployable, and simultaneous dual-gene detection of SARS-CoV-2 RNA via modified RT–RPA. Cell Discov..

[9] Huang W., Lin D., Wang C., Bao C., Zhang Z., Chen X., Zhang Z., Huang J. (2021). The determination of release from isolation of COVID-19 patients requires ultra-high sensitivity nucleic acid test technology. J. Infect..

[10] Behrmann O., Bachmann I., Spiegel M., Schramm M., El Wahed A.A., Dobler G., Dame G., Hufert F.T. (2020). Rapid detection of SARS-CoV-2 by low volume real-time single tube reverse transcription recombinase polymerase amplification using an exo probe with an internally linked quencher (exo-IQ). Clin. Chem..

[11] Basu A., Zinger T., Inglima K., Woo K., Atie O., Yurasits L., See B., Aguero-Rosenfeld M.E. (2020). Performance of Abbott ID Now COVID-19 Rapid Nucleic Acid Amplification Test Using Nasopharyngeal Swabs Transported in Viral Transport Media and Dry Nasal Swabs in a New York City Academic Institution. J. Clin. Microbiol..

[12] Harrington A., Cox B., Snowdon J., Bakst J., Ley E., Grajales P., Maggiore J., Kahn S. (2020). Comparison of Abbott ID Now and Abbott m2000 Methods for the Detection of SARS-CoV-2 from Nasopharyngeal and Nasal Swabs from Symptomatic Patients. J. Clin. Microbiol..

[13] Dunbar S., Das S. (2019). Amplification chemistries in clinical virology. J. Clin. Virol..

[14] Ménová P., Raindlová V., Hocek M. (2013). Scope and Limitations of the Nicking Enzyme Amplification Reaction for the Synthesis of Base-Modified Oligonucleotides and Primers for PCR. Bioconjug. Chem..

[15] Morris S.R., Bristow C.C., Wierzbicki M.R., Sarno M., Asbel L., French A., Gaydos C.A., Hazan L., Mena L., Madhivanan P. (2021). Performance of a single-use, rapid, point-of-care PCR device for the detection of Neisseria gonorrhoeae, Chlamydia trachomatis, and Trichomonas vaginalis: A cross-sectional study. Lancet Infect. Dis..

[16] Calame A., Mazza L., Renzoni A., Kaiser L., Schibler M. (2021). Sensitivity of nasopharyngeal, oropharyngeal, and nasal wash specimens for SARS-CoV-2 detection in the setting of sampling device shortage. Eur. J. Clin. Microbiol. Infect. Dis..

[17] Clopper C.J., Pearson E.S. (1934). The Use of Confidence or Fiducial Limits Illustrated in the Case of the Binomial. Biometrika.

[18] Essaidi-Laziosi M., Geiser J., Huang S., Constant S., Kaiser L., Tapparel C. (2020). Interferon-Dependent and Respiratory Virus-Specific Interference in Dual Infections of Airway Epithelia. Sci. Rep..

[19] Corman V.M., Landt O., Kaiser M., Molenkamp R., Meijer A., Chu D.K., Bleicker T., Brünink S., Schneider J., Schmidt M.L. (2020). Detection of 2019 novel coronavirus (2019-nCoV) by real-time RT-PCR. Euro Surveill..

[20] Artesi M., Bontems S., Göbbels P., Franckh M., Maes P., Boreux R., Meex C., Melin P., Hayette M.-P., Bours V. (2020). A Recurrent Mutation at Position 26340 of SARS-CoV-2 Is Associated with Failure of the E Gene Quantitative Reverse Transcription-PCR Utilized in a Commercial Dual-Target Diagnostic Assay. J. Clin. Microbiol..

[21] CDC (2020). Information for Laboratories about Coronavirus (COVID-19). https://www.cdc.gov/coronavirus/2019-ncov/lab/guidelines-clinical-specimens.html.

[22] Bullard J., Dust K., Funk D., Strong J.E., Alexander D., Garnett L., Boodman C., Bello A., Hedley A., Schiffman Z. (2020). Predicting infectious SARS-CoV-2 from diagnostic samples. Clin. Infect. Dis. Off. Publ. Infect. Dis. Soc. Am..

[23] Porte L., Legarraga P., Vollrath V., Aguilera X., Munita J.M., Araos R., Pizarro G., Vial P., Iruretagoyena M., Dittrich S. (2020). Evaluation of a novel antigen-based rapid detection test for the diagnosis of SARS-CoV-2 in respiratory samples. Int. J. Infect. Dis..

[24] Scohy A., Anantharajah A., Bodéus M., Kabamba-Mukadi B., Verroken A., Rodriguez-Villalobos H. (2020). Low performance of rapid antigen detection test as frontline testing for COVID-19 diagnosis. J.Clin. Virol..

[25] Diao B., Wen K., Zhang J., Chen J., Han C., Chen Y., Wang S., Deng G., Zhou H., Wu Y. (2021). Accuracy of a nucleocapsid protein antigen rapid test in the diagnosis of SARS-CoV-2 infection. Clin. Microbiol. Infect..

[26] Mak G.C.K., Lau S.S.Y., Wong K.K.Y., Chow N.L.S., Lau C.S., Lam E.T.K., Chan R.C.W., Tsang D.N.C. (2021). Evaluation of rapid antigen detection kit from the WHO Emergency Use List for detecting SARS-CoV-2. J. Clin. Virol..

[27] Weitzel T., Legarraga P., Iruretagoyena M., Pizarro G., Vollrath V., Araos R., Munita J.M., Porte L. (2020). Head-to-head comparison of four antigen-based rapid detection tests for the diagnosis of SARS-CoV-2 in respiratory samples. bioRxiv.

[28] Gao X., Sun B., Guan Y. (2019). Pullulan reduces the non-specific amplification of loop-mediated isothermal amplification (LAMP). Anal. Bioanal. Chem..

[29] Karthik K., Rathore R., Thomas P., Arun T.R., Viswas K.N., Dhama K., Agarwal R.K. (2014). New closed tube loop mediated isothermal amplification assay for prevention of product cross-contamination. MethodsX.

[30] Hsieh K., Mage P.L., Csordas A.T., Eisenstein M., Soh H.T. (2014). Simultaneous elimination of carryover contamination and detection of DNA with uracil-DNA-glycosylase-supplemented loop-mediated isothermal amplification (UDG-LAMP). Chem. Commun..

[31] Wang D.-G., Brewster J.D., Paul M., Tomasula P.M. (2015). Two Methods for Increased Specificity and Sensitivity in Loop-Mediated Isothermal Amplification. Molecules.

