# Supplementary material for: Analytical Evaluation of Visby Medical RT-PCR Portable Device for Rapid Detection of SARS-CoV-2
Source: Diagnostics (Basel). 2021 Apr 29;11(5):813. doi: 10.3390/diagnostics11050813 (PMC8146445; doi:10.3390/diagnostics11050813)
Supplement: Supplementary file 1 [file diagnostics-11-00813-s001.zip › diagnostics-1156305-supplementary.pdf]

Device No.	Cobas CT E gene	VISBY Result	VISBY Repetition	Concordance	Cobas Repetition	GeneXpert N / E gene
1	16	POS		C		
2	17	POS		C		
3	17	POS		C		
4	18	POS		C		
5	18	POS		C		
6	19	POS		C		
7	19	POS		C		
8	19	POS		C		
9	19	POS		C		
10	19	POS		C		
11	20	POS		C		
12	21	POS		C		
13	21	POS		C		
14	21	POS		C		
15	21	INV	POS	C		
16	21	POS		C		
17	21	POS		C		
18	22	POS		C		
19	22	POS		C		
20	22	POS		C		
21	23	POS		C		
22	23	POS		C		
23	23	POS		C		
24	24	POS		C		
25	25	POS		C		
26	25	POS		C		
27	25	POS		C		
28	27	POS		C		
29	29	POS		C		
30	29	POS		C		
31	30	POS		C		
32	31	POS		C	31	
33	31	NEG	NEG	NC	31	
34	33	POS		C	35	
39	27	POS		C		
40	17	POS		C		
41	16	POS		C		
42	19	POS		C		
43	26	POS		C		
44	17	POS		C		
45	17	POS		C		
46	32	POS		C	33	
47	22	POS		C		
48	18	POS		C		
49	25	POS		C		
50	17	POS		C		
51	26	POS		C		
52	23	POS		C		
53	25	POS		C		
54	21	POS		C		
55	20	POS		C		
56	21	POS		C		
57	26	POS		C		
58	34	POS		C	34	
59	32	POS		C	34	
60	34	NEG		NC	34	37.8 / 34.5
61	0	NEG		C		
62	0	NEG		C		
63	0	NEG		C		
64	0	NEG		C		
65	0	NEG		C		
75	32	NEG		NC		35.1 / 31.9
76	33	POS		C		
77	34	POS		C		
79	24	POS		C		
83	34	POS		C		
84	0	NEG		C		
85	0	NEG		C		
86	0	NEG		C		
87	0	NEG		C		
88	0	NEG		C		
89	0	NEG		C		
90	0	NEG		C		
91	0	NEG		C		
92	0	NEG		C		
93	0	NEG		C		
95	0	NEG		C		
96	0	NEG		C		

Supplemental Table 1. Positive and negative RT-qPCR Cobas samples tested on RT-PCR Visby diagnostic device. **C** = corncordant result; **NC** = Non-concordant results. INV= invalid result.
